# Practical Notes on Diagnosis and Treatment

**Published:** 1910-08-13

**Authors:** 


### Practical Notes on Diagnosis and Treatment.

**Tubal Pregnancy.**

The diagnosis of tubal pregnancy before hæmorrhage has occurred is impossible.—*Dr. G. Herman.*

**Empyema.**

Remember that an important guide to the localisation of empyemata is local tenderness. When there is uncertainty it is a good plan to push the finger against the ribs and insert the needle at the tenderest spot.—*Dr. H. White.*

**Enteric Fever.**

The temperature of convalescents, especially from typhoid, is a very sensitive thing; a very slight cause seems sufficient to raise it. A common cause is constipation; others are errors of diet and the excitement of visitors.—*Dr. Ormerod.*

**Etiology of Gout.**

There is much evidence in favour of the view that gout and rheumatism arise as a septicæmic or sapræmic condition associated with a source of infection in the mouth. I have never failed to find pyorrhœa in an adult who has had rheumatism or gout.—*Dr. Wynn Wirgman.*

**Intestinal Obstruction.**

If a patient has obstruction which will not even let flatus pass, and you give him purgatives which cause a large watery flow into the intestine, you will distend the bowel in thirty-six hours more dangerously than it would distend itself in a week. The only thing you are justified in giving is an enema.—*Mr. A. Bowlby.*

**Fractured Base.**

With regard to lumbar puncture I do not look upon this method as of any use in the treatment of injuries to the head. From a diagnostic point of view the presence of blood in the cerebro-spinal fluid, as withdrawn by lumbar puncture, suggests the presence of intradural hæmorrhage, nothing more.—*Mr. L. B. Rawling.*

**Operation for Graves' Disease.**

Bad acute cases of typical Graves' disease should rarely be operated upon. If operation is undertaken it should only be done after a prolonged course of medical treatment. Operation of any kind in these cases is usually exceedingly dangerous to life, and the prospect of complete cure is by no means very hopeful.—*Mr. James Berry.*

**Migraine.**

The association of epilepsy with migraine is of considerable interest. It occasionally happens that periodical attacks of migraine are replaced by epilepsy, the migraine then ceasing.—*Dr. A. E. Russell.*

**Phagocytosis.**

Phagocytes are destroyed by quinine, and as it is known that the action of quinine in lowering the temperature is chiefly due to the destruction of leucocytes it will be realised that quinine is contra-indicated in all bacterial infections.—*Dr. E. von Ofenheim.*

**Uterine Carcinoma.**

Carcinoma of the body of the uterus is pre-eminently a disease of advanced life. The majority of cases occur between the ages of fifty and sixty. There is a large percentage of sterile women in a series of cases of carcinoma of the body of the uterus.—*Dr. A. N. Lewers.*

**Gonorrhœal Rheumatism.**

It must be remembered that gonorrhœal rheumatism is not necessarily a lesion of acute gonorrhœa; it may come on years after, when there may be no trace of any discharge at all, either gleet or purulent discharge.—*Dr. Herringham.*

**Neurasthenia and Graves' Disease.**

I have had cases of neurasthenia and Graves' disease under my care which have been associated with oral sepsis, and in which the relief of the latter condition has been attended by an improvement in both the other two conditions. It is admitted that neurasthenic symptoms may be a consequence of the thyroid derangement we call Graves' disease, but I think it likely that in many cases the toxæmic condition underlying both these diseases is the oral sepsis present.—*Dr. T. D. Savill.*

**Lithium in Gout.**

The salts of lithium are popular remedies for gout, but they are of doubtful efficacy in medicinal doses. They are excreted by the kidneys, it is true, but they exert no greater diuretic effect than would be produced by an equal quantity of common salt. They act as solvents for uric acid, but only in such a degree of concentration as would produce toxic symptoms. It may be safely said that lithia is of no value in the treatment of gout.—*Dr. Wm. Murrell.*

